# Varying the Polishing Protocol Influences the Color Stability and Surface Roughness of Bulk-Fill Resin-Based Composites

**DOI:** 10.3390/jfb12010001

**Published:** 2020-12-22

**Authors:** Filipa Freitas, Teresa Pinheiro de Melo, António HS Delgado, Paulo Monteiro, João Rua, Luís Proença, Jorge Caldeira, Ana Mano Azul, José João Mendes

**Affiliations:** 1 Clinical Research Unit (CRU), Centro de Investigação Interdisciplinar Egas Moniz (CiiEM), Instituto Universitário Egas Moniz (IUEM), 2829-511 Almada, Portugal; filipamdfreitas@gmail.com (F.F.); tpmelo@egasmoniz.edu.pt (T.P.d.M.); pmonteiro@egasmoniz.edu.pt (P.M.); jrua@egasmoniz.edu.pt (J.R.); lproenca@egasmoniz.edu.pt (L.P.); jcaldeira@egasmoniz.edu.pt (J.C.); amanozul@egasmoniz.edu.pt (A.M.A.); jmendes@egasmoniz.edu.pt (J.J.M.); 2 Department of Biomaterials and Tissue Engineering, Royal Free Hospital, UCL Eastman Dental Institute, London NW3 NPF, UK; 3 Quantitative Methods for Health Research (MQIS), CiiEM, Instituto Universitário Egas Moniz (IUEM), 2829-511 Almada, Portugal; 4 UCIBIO and LAQV Requimte, Faculdade de Ciências e Tecnologias, Universidade Nova de Lisboa, 2829-516 Almada, Portugal

**Keywords:** color stability, dental composites, methacrylate-based resin, surface roughness

## Abstract

Surface properties of composites such as roughness and color impact periodontal health and aesthetic outcomes. Novel bulk-fill composites with improved functionality are being introduced and, in light of the existing variety of finishing/polishing procedures, research of their surface properties is warranted. Sixty discs were prepared from bulk-fill composites (Filtek™ Bulk Fill Posterior Restorative and Fill-Up™) and incremental-fill Filtek™ Z250. They were further divided according to different polishing procedures (*n* = 5): three multi-step polishing procedures or finishing with a bur (control). Surface roughness (Ra) was measured using an atomic force microscope (The AFM Workshop TT-AFM). A spectrophotometer (Spectroshade Micro Optic) was used to determine color stability, after exposure to a coffee solution. Data were analyzed using two-way MANOVA (significance level of 5%). Resin composite type, polishing procedure, and their interaction had a statistically significant effect on surface roughness (*p* < 0.001) and color change (*p* < 0.001). Fill-Up™ exhibited the highest surface roughness and greatest color change. Differences in color change were statistically significant (*p* < 0.001). Filtek™ Bulk Fill registered the lowest surface roughness and color change, after the three-step polishing procedure. Both parameters were significantly correlated (ρ = 0.754, *p* < 0.001) and found to be material dependent and polishing-procedure dependent. Higher surface roughness relates to greater color changes.

## 1. Introduction

Resin composites are widely used in dentistry due to their optical and mechanical properties, comparable to those of enamel and dentin, making them a reliable and decent biomimetic replacement [1,2]. Nonetheless, these materials feature shortcomings which include poor color stability, susceptibility to wear, leakage, and polymerization shrinkage [3]. 

Composites also require maintenance when it concerns their surface finish. Recent evidence has proven there is an acceptable surface roughness range to which bacterial adhesion and biofilm formation is limited [4]. Thus, compliance with this range has to be achieved clinically. This is known to directly impact periodontal health outcomes and can lead to localized inflammation and gingival recession [5,6]. Increased plaque accumulation aggravates periodontal tissue inflammation and reduces clinical longevity [7,8]. Owing to this, special care, undertaking appropriate and careful finishing/polishing, is required in periodontal patients. Inadequate surface properties of restorative materials may aggravate periodontitis, which consequently compromises other systemic disorders, as reported in recent studies [9,10]. 

Other than periodontal issues, caries recurrence may also be avoided by optimizing surface roughness [7]. Furthermore, an optimal surface finish is required if restorative materials are aiming for biomimetism. Enamel has a low surface roughness when polished (0.02–0.05 µm), and this is linked to its optical properties, affecting light reflection [11].

Dental composites are prone to discoloration, owing to intrinsic factors related to the materials’ properties, such as chemical changes following reactivity, matrix constituents (type of monomers, interaction between resin-filler phase), and photo-initiators [12]. Extrinsic factors resulting from exogenous sources such as food, drinks, and smoking habits also play a role. Severe discoloration can compromise the appearance of the restoration and is regarded as an aesthetic failure [13]. Additionally, the surface roughness of a restoration influences not only bacterial colonization and dental plaque formation but also parameters such as color stability, wear, and overall aesthetic appearance. Thus, finishing and polishing procedures are paramount to not only achieve acceptable aesthetics but also to guarantee the longevity of the composite restoration [4,14].

Resin composites are a complex mixture of methacrylate-based monomers with an inorganic phase, which, when polymerized, stiffens due to an increase in the cross-linking of the polymer chain. In the post-gel contraction phase, interfacial defects occur due to the shrinkage-related strain of the material [15,16]. Gap formation may increase the potential for post-operative sensibility and may also ultimately lead to microleakage and recurrent caries. This stress that is generated depends on the type of resin monomers involved, filler technology, gel point, C-factor, elastic modulus of the material, and also curing technique and degree of conversion [16]. A lower degree of conversion of the monomers also provides inferior mechanical properties and leads to greater color change and degradation [1,17].

In order to overcome limited curing depth in large and deep cavities [18], reducing time-consuming techniques, and other polymerization drawbacks, a new class of resin composites was introduced—the bulk-fill composites [1,19]. The advantage of this new material, which has a higher translucency, is that it can be placed in a 4 mm thickness increment in one easy step. This avoids the adverse effect of polymerization shrinkage and, due to differences in the composition related to filler size, distribution, and initiators, the degree of conversion may also be improved. Research has shown, however, that these advantages may not always be present in comparison to traditional incremental fill [18,20,21]. Bulk-fill resin composites also exhibit different optical properties due to differences in their composition and filler content [22]. These differences should affect their color stability and surface roughness. 

Since many different polishing protocols exist, and different commercial options of novel bulk-fill resin composites are available, it is important to find out whether these materials can achieve results comparable to traditional composites, and which finishing/polishing protocols should be recommended to minimize negative outcomes. Therefore, the aim of this study was to evaluate the surface roughness of two bulk-fill resin composites after being submitted to different clinically reproducible polishing procedures, as well as their color stability after immersion in a coffee solution, comparing them to traditional incremental-fill hybrid resin composite. This provides insight into the surface properties of contemporary bulk-fill resin composites after finishing/polishing and staining challenges. It also gives information on the best finishing/polishing combination that leads to lower surface roughness and higher stain resistance. Furthermore, the interdependency and correlation of both factors (roughness and color stability), according to the composites and polishing protocols that were evaluated, were also studied. 

## 2. Materials and Methods 

### 2.1. Materials and Sample Preparation

A total of sixty discs, twenty per group, were prepared from three commercial resin composites: Filtek™ Bulk Fill Posterior Restorative, Fill-Up™, and Filtek™ Z250 (Table 1). Each disc was prepared using a stainless-steel mold—12-mm diameter, 2-mm thickness dimensions for Filtek™ Z250, or 4-mm thickness for Filtek™ Bulk Fill and Fill-Up™. Specimens were polymerized with Elipar Deep-cure-S LED curing-light (3M ESPE, St. Paul, MN, USA), at zero distance, separated by an acetate sheet, using a 4-point overlapping irradiation cycle, for 20 s, on both top and bottom surfaces, in accordance with ISO 4049:2019. The polymerization intensity was set to ensure at least 900 mW/cm^2^ and was monitored regularly, after every four exposures, using a radiometer—Model 100 Curing Radiometer (Demetron Research Corporation, Dunbury, CT, USA). Specimens (*n* = 20) within each resin composite group were randomly allocated to each one of the four subgroups, using a random generated sequence (computer generated), formed according to the polishing protocols (P1-P4). A control (P4) was included in each of the groups, where surface finishing was achieved by means of a diamond bur. A total of 12 groups (*n* = 5) were formed. 

### 2.2. Polishing Protocols

The instruments and procedures used on each polishing protocol are listed in Table 2. The different finishing and polishing procedures were employed according to the instructions provided by the manufacturer, which are included in Table 2. The procedures were carried out by the same operator, using a low-speed contra-angle handpiece (DPS Line M4, KMD, Europe, Bilbao-Vizcaya, Spain), except for the polishing brush SHP Soft Bristle Brush (DPS Line M4, KMD Europe, Bilbao-Viscaya, Spain) which was used with a low-speed surgical handpiece (DPS Line M4, KMD, Europe, Bilbao-Vizcaya, Spain). Each polishing instrument was used for a continuous 30 s, with water cooling, except for polishing procedure 2 and the final stage of polishing procedure 3 (Table 2). Manufacturer’s instructions were followed for rpm and contact pressure. After each polishing procedure, the samples were rinsed for 10 s and air dried. The specimens were stored in distilled water at 37 °C for 24 h in an incubator oven.

### 2.3. Surface Roughness Determination

All specimens were subject to surface roughness evaluation using an atomic force microscope—TT-AFM (The AFM Workshop, Signal Hill, California, USA). Both deflection and height-mode images were obtained at a fixed scan rate of 0.4 Hz, using a vibrating mode, and a resolution of 512 × 512 pixels. AFM images, obtained with MountainsMap^®^ Premium software, Version 7.3 (Digital Surf, Besançon, France), were acquired from the central region of each sample, with 40 × 40 µm dimensions. These images were used to calculate the average surface roughness (Ra), in nm, which was measured using Gwyddion software, Version 2.45 (CMI, Prague, Czech Republic). The software allows the image to be divided into 16 different sections (with 10 × 10 µm in size) in order to obtain the average value of surface roughness for each section. Thus, in total, 16 image sections were obtained for each sample and used to calculate the mean surface roughness value of the specimen.

### 2.4. Color Stability Determination

To determine the color of the specimens, a digital spectrophotometer Spectroshade Micro Optic (MHT S.p.A., Arbizzano di Negrar, Italy) was used according to ISO/TR 28642:2016 and the CIELAB scale. A measurement was obtained for the individual color coordinates (L*, a*, and b*), which represent lightness value, red/green value, and blue/green, respectively. The measurement was performed twice for each specimen and the device was recalibrated after each measurement. A black box for sample positioning, with standardized site, angle, and surrounding illumination was used as a background during measurements. Following the baseline measurements, the specimens were immersed in a cyclic coffee solution replaced every 24 h, prepared with hot water and instant coffee (50 g of coffee, 500 mL of water) and stored in vials, for a total period of 14 days, following Barakah and Taher’s (2014) protocol [23]. They were kept at a constant temperature of 37 °C in an incubator. Before the final color measurement, the specimens were rinsed with distilled water for one minute followed by air drying. To assess color change, the following CIE (International Comission on Illumination) formula was used to determine color differences: ΔE = [(ΔL*)^2^ + (Δa*)^2^ + (Δb*)^2^]^1/2^, where Δ represents the variation between the initial and final measurements (before and after the 14-day immersion period) for each coordinate, and for color overall—represented by ΔE. This is in accordance with ISO/CIE 11664-4:2019.

### 2.5. Statistical Analysis

Data analysis was performed using IBM SPSS Statistics version 24.0 for Windows (IBM, Armonk, NY, USA). Descriptive statistics as mean and standard deviation were calculated. Population means were estimated by calculating 95% confidence intervals (95% CI). A statistical inference analysis was carried out by using a factorial, two-way MANOVA, considering the following fixed factors: polishing procedure and resin composite type. Surface roughness and color change were considered as dependent variables within the model. Prior to the factorial analysis, the MANOVA model assumptions were validated and a bivariate analysis, by using the Spearman rank correlation coefficient (ρ), was conducted in order to assess the correlation between surface roughness and color change values. A multiple comparison analysis was performed by Tukey HSD post-hoc test. Estimated effect sizes, within the factorial model, were achieved by calculating the partial eta-squared coefficient (ƞ^2^_p_). The level of statistical significance was set at 5% in all inferential analyses.

## 3. Results

### 3.1. Surface Roughness and Topography Imaging

**Figure 1:**
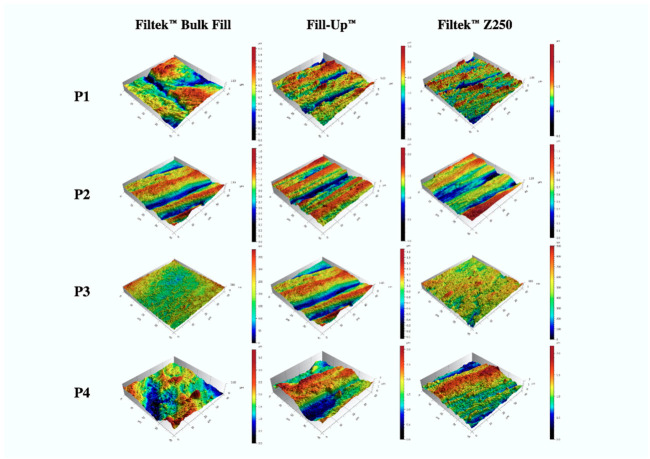
3D surface topography according to AFM imaging (40 × 40 µm).

Mean surface roughness (Ra) values and correspondent 95% confidence interval (CI) for mean for resin composites tested after different finishing and polishing procedures are shown in Table 3. Higher surface roughness values were obtained for Fill-Up™ with polishing protocols P4 (control) (Ra = 328.6 (±27.5) nm) and P1 (Ra = 304.5 (±31.0) nm), respectively. Conversely, lower values were obtained for the groups submitted to the polishing protocol P3 with Filtek™ Bulk Fill (Ra = 40.8 (±18.7) nm) and Filtek™ Z250 (Ra = 68.1 (±15.2) nm). Three-dimensional AFM imagery (with 40 × 40 µm dimensions), representative of the surface topography of the different resin composites and polishing procedures combinations, is depicted in Figure 1. 

### 3.2. Color Stability

Mean color change (∆E) values and correspondent 95% CI for mean for resin composites tested after different finishing and polishing procedures are shown in Table 4. Greater color change was obtained for Fill-Up™ (∆E = 14.6 (±0.4) %) and Filtek™ Bulk Fill (∆E = 13.2 (±0.6) %), with polishing protocol P4 (control), while the least changes in color were observed for resin composite Filtek™ Z250 with polishing protocol P3 (∆E = 7.2 (±0.4) %).

### 3.3. Correlation and Factorial Analysis

Overall, surface roughness and color change were found to be significantly correlated, with greater color differences observed for higher mean surface roughness values (ρ = 0.754, *p* < 0.001). Results for the analysis of the mean surface roughness and color change among groups, as a function of the experimental factors, resin composite, and polishing procedure, by using a factorial two-way MANOVA, are presented in Table 5. Resin composite type, polishing procedure, and the interaction between the two factors had a statistically significant effect on both surface roughness and color change. Correspondent estimated mean values are shown in Table 6 (resin composite type) and Table 7 (polishing procedure).

When considering surface roughness, the estimated effect size was found to be very strong both for the resin composite factor (*p* < 0.001, ƞ^2^*_p_* = 0.889) and polishing procedure (*p* < 0.001, ƞ^2^*_p_* = 0.847). Interaction between resin composite type and polishing procedure was also shown to be relevant (*p* < 0.001, ƞ^2^*_p_* = 0.504). Resin composite Fill-Up™ had significantly greater (*p* < 0.001, Tukey HSD test) surface roughness estimated mean values (Ra = 266.2 nm) when compared to Filtek™ Bulk Fill (Ra = 135.5 nm) and Filtek™ Z250 (Ra = 133.6 nm). No significant difference between Filtek™ Bulk Fill and Filtek™ Z250 was observed (*p* = 0.965, Tukey HSD test). Regarding the polishing procedure effect, significant differences were found among the polishing protocols, with higher surface roughness estimated mean values for polishing protocol P4 (control) (Ra = 253.2 nm), followed by P1 (Ra = 186.0 nm), P2 (Ra = 165.3 nm), and P3 (Ra = 109.2 nm). Significant differences were noted between P4 (control) and the other polishing procedures (*p* < 0.001, Tukey HSD test). Significant differences were also found between P3 and the other polishing protocols (*p* < 0.001, Tukey HSD test). No significant differences were noted between polishing protocols P1 and P2 (*p* = 0.108, Tukey HSD test).

When considering color change, the estimated effect size was higher for the polishing procedure factor (*p* < 0.001, ƞ^2^*_p_* = 0.891) than for resin composite type (*p* < 0.001, ƞ^2^*_p_* = 0.699), indicating a more important effect for the first factor, regarding color change. The interaction effect between resin composite type and polishing procedure factors was also noted to be relevant (*p* = 0.005, ƞ^2^*_p_* = 0.309). The estimated difference between color change among the three resin composites was statistically significant (*p* < 0.001, Tukey HSD test). Filtek™ Z250 (∆E = 9.6%) had less color change, followed by Filtek™ Bulk Fill (∆E = 10.5%) and Fill-Up™ (∆E = 11.9%). Additionally, when considering the polishing procedure effect, significant differences were noted among all four polishing protocols (*p* < 0.001, Tukey HSD test). The estimated color change difference for the control polishing protocol P4 (∆E = 13.4%) was found to be significantly higher than P1 (∆E = 10.8%), P2 (∆E = 9.5%), and P3 (∆E = 8.8%).

When comparing the effect of both factors, polishing procedure and resin composite type, on surface roughness and color change, it is possible to infer that polishing procedure plays a major role in both parameters; however, it has a bigger influence on the first one. Interaction effect between resin composite type and polishing procedure factors was found to be more important for surface roughness than for color change (ƞ^2^*_p_* = 0.504 vs. 0.309).

## 4. Discussion

This study highlights the impact of both material composition and different finishing/polishing procedures on the surface properties of contemporary resin composites, providing evidence that bulk-fill materials can achieve comparable properties to traditional incremental fill. Improper finishing and polishing procedures can compromise the clinical performance of the restoration due to increased wear rates and susceptibility to plaque formation [24]. Past literature has pointed out that finishing procedures in resin composites should always be succeeded by fine polishing [4]. The smoothness of the restoration is influenced by the type of resin composite. It largely depends on the fillers (their type, shape, size, and distribution), the organic matrix, its interface. and the finishing and polishing procedures carried out [25,26]. Not only is smoothness important for functional aspects but it is also a requirement for good esthetic outcomes. A glossy and smooth surface is usually an indication of a well-polished restoration [27]. In this study, bulk-fill composite Fill-Up™ registered higher surface roughness values compared to Filtek™ Bulk Fill and Filtek™ Z250. These results can be explained by differences in particle size. It is well established that resin composites with smaller particle sizes facilitate higher gloss and lower surface roughness values after sequential polishing protocols [25], as is the case with Filtek™ Z250. The use of a finer filler size results in less interparticle spacing, which in turn protects the resin matrix and reduces filler plucking [28,29].

Filtek™ Bulk Fill and Filtek™ Z250 have higher filler loading (58.4% and 60%, respectively), compared to Fill-Up™ (49%), which also correlates with lower surface roughness values. According to previous studies, the surface roughness is lowered by decreasing the filler size and increasing the overall filler content [30,31]. Filtek™ Bulk Fill can match the traditional, incremental-fill Filtek™ Z250 in surface roughness values, as no differences were found in the estimated means pooled from the different polishing procedures. Bulk-fill composites feature changes in their organic matrix, filler content, and/or size [30,32]. Usually, manufacturers increase the depth of cure by enhancing the material’s translucency. This is achieved by decreasing the filler amount or increasing the size of the fillers [33]. This is also accomplished by modifications in the initiator systems.

The surface roughness was found to be mainly influenced by the type of resin composite, as stated above, but also by the polishing procedure used. This is in accordance with many previous findings [34,35,36]. Smoother surfaces were achieved with the experimental polishing protocols, when compared to the control group, where the samples were finished with an extra-fine bur. According to the literature, the quality of the final polished surface is dependent upon the flexibility of the instrument, its geometry, and overall hardness of the particles [37].

Authors lack standardization in what concerns recommended minimum surface roughness values. Some authors report a threshold of 200 nm as the minimum recommended, while others go up to as high as 1440 nm [4,38]. A recent systematic review on the subject highlights that instead of a threshold, a range of roughness exists, in which polishing is considered decent for biological and physical factors [4]. In this study, all surface roughness values measured after the experimental polishing procedures fell below 200 nm, suggesting an unfavorable surface for bacterial attachment, which is what is predicted below this threshold [4,38,39]. Surface roughness is also critical in restorations that have subgingival margins, or class V restorations of root caries [6]. The tooth attachment may be compromised if periodontal pockets develop, and hygiene is difficult. Recent research has been devoted to the development of antibacterial composites against periodontal pathogens able to solve this problem [40]. Furthermore, the accumulation and permanence of biofilm close to gingival margins may result in recession around aged and roughened surfaces [41].

In this study, coffee was used as a staining agent due to its frequent consumption in daily life. Filtek™ Z250, which had the lower surface roughness values, showed higher color stability, and the Fill-Up™ resin composite which had higher surface roughness values, showed the opposite. These results are in accordance with studies that reported that the structure of a resin composite and the characteristics of the filler particles have a direct impact on surface roughness and susceptibility to extrinsic staining [24], since rougher surfaces are able to mechanically retain more stains [42].

When it comes to color stability and staining of composites, not only the chemistry of the organic matrix and fillers is important, but also the finishing/polishing procedures that are chosen [22,42]. In fact, in this study, color stability was more dependent upon the finishing/polishing system used than the type of material. This can be explained by what was mentioned above, as stain resistance increases when surface roughness decreases [42,43]. Alterations in the topography of the composite’s surface, resulting from abrasion of the organic matrix and loss of filler particles at the surface, lead to an increase in roughness and subsequent decrease of surface gloss. Finishing/polishing procedures are able to expose the fillers by smoothening the surface, ultimately reinstating favorable optical properties [27].

The organic matrix and its constituent monomers directly influence color stability due to characteristics such as degree of conversion and hydrophilicity. Bulk-fill Fill-Up™ showed the lowest color stability out of all the materials tested in this study. The greater color change registered with bulk-fill Fill-Up™ might be due to the higher content of resin matrix when compared to the alternatives. This results in greater susceptibility to hydrolytic degradation and water sorption [44]. Fill-Up™ is also a dual-cure resin composite. According to the literature, dual-cure resin composites are more prone to color changes compared with light-cured resin composites [3]. Some of the commercial bulk-fill resin composites feature a higher content of organic matrix than conventional composites. Even though the expected degree of conversion is higher, the greater content of organic matrix in bulk-fill materials may promote further staining. A monomeric matrix containing a substantial amount of thriethylene glycol dimethacrylate (TEGDMA) can lead to higher water sorption and consequently, pigmentation of the material [23,45].

In this study, in order to achieve greater color stability and smoother surfaces, the multi-step polishing procedure featuring Diatech Polishing Plus two-step system followed by DiaShine intraoral polishing paste and Brushine brush was the one which performed best. Diamond abrasive particles, specifically in the form of a vehicle, such as a polishing paste, which facilitates their dispersion, are instrumental in the development of a surface with a relatively low roughness associated with it, as shown by Lopes et al. (2018) [39]. Since this multi-step protocol featured more steps than the alternative experimental protocols carried out in this study, it required a total polishing time that was higher than in the other methods. This may also have contributed to the color stability and surface roughness results, and longer polishing protocols with different steps are, thus, recommended.

## 5. Conclusions

According to the results obtained in this laboratory study and within its limitations, the following conclusions can be drawn: the resin composite Fill-Up™ had the highest associated surface roughness and lowest color stability, which may be explained by differences in its chemical composition and polymerization mechanisms. Surface roughness and color stability depend significantly on the type of resin composite used and polishing procedure. For the materials evaluated, color stability seems to depend more upon the finishing/polishing procedure than the material chemistry, while for surface roughness outcomes, both the finishing/polishing system and material chemistry showed strong effect sizes. There is a significant correlation between surface roughness and color stability, where higher surface roughness values correspond to greater color differences. A finishing and polishing protocol with carefully planned steps, taking the necessary time, will improve the surface properties of the resin composite, leading to durable outcomes. Bulk-fill resin composites are able to achieve surface properties comparable to incremental-fill composites.

## Figures and Tables

**Table 1:** Material properties and information (derived from the manufacturer’s information and safety datasheets).

	Filtek™ Bulk Fill Posterior Restorative	Fill-Up™	Filtek™ Z250
**Organic Matrix**	AUDMA (10%–20%) DDDMA (<10%) UDMA (1%–10%) AFM	TMPTMA (10%–15%) UDMA (10%–15%) Bis-GMA (5%–10%) TEGDMA (1%–5%)	UDMA (1%–10%) Bis-EMA (1%–10%) Bis-GMA (1%–10%) TEGDMA (1%–5%)
**Filler Type and Size**	Silica nanoparticles (20 nm), Zirconia nanoparticles (4–11 nm), and trifluoride ytterbium (100 nm)	Silica particles and coated zinc oxide (1%–5%) Range 0.1–5 µm	Silica, aluminum oxide, andzirconia particles Range 0.01–3.5 µm
**Filler load (wt. %)** **(vol %)**	76.5 58.5	65 49	82 60
**Color**	A3	Universal (A2-A3)	A3
**Manufacturer**	3M™ ESPE™ (St. Paul, MN, USA)	Coltène-Whaledent (Altstätten, Switzerland)	3M™ ESPE™ (St. Paul, MN, USA)
**Batch**	N726218	G14043	N726150
**Instructions**	4 mm thickness increments 40 s polymerization	Dual cured resin Any thickness increments. 10 s polymerization	Increments with a maximum thickness of 2.5 mm. 20 s polymerization

AFM: stress-relieving monomer; AUDMA: aromatic urethane dimethacrylate; Bis-EMA: Bisphenol A diglycidyl methacrylate ethoxylated; Bis-GMA: Bisphenol A glycidyl dimethacrylate; DDDMA: 1,12 dodecane-dimethacrylate; TEGDMA: thriethylene glycol dimethacrylate; TMPTMA: Trimethylolpropane trimethacrylate; UDMA: urethane dimethacrylate.

**Table 2:** Polishing protocols and laboratory procedure.

	Material	Supplier	Procedure
Polishing protocol No. 1 (P1)	Sof-lex™ pre-polishing spiral (yellow) Batch: N508797	3M™ ESPE™ (St. Paul, MN, USA)	Pre-polishing with rubber spiral coated with aluminum oxide, followed by diamond polishing spiral wheel.
	Sof-lex™ Diamond Polishing Spiral (purple) Batch: N782180	3M™ ESPE™ (St. Paul, MN, USA)	
Polishing protocol No. 2 (P2)	DIATECH^®^ Shape Guard Composite Polishing Plus Kit Batch: G97378	Coltène-Whaleden, (Altstätten, Switzerland)	Pre-polishing with purple diamond polish spiral Comprepol Plus (23SG14RA) followed by blue diamond spiral Composhine Plus (24SG14RA).
Polishing protocol No. 3 (P3)	DIATECH^®^ Composite Polishing Plus Kit Batch: G84512	Coltène-Whaledent, (Altstätten, Switzerland)	Pre-polishing with diamond rubber cup—purple (2311RA) followed by blue diamond spiral (2411RA). Diamond polishing paste Intra Oral Diashine * applied with goat hairbrush (SHP Soft Bristle Brush). Finished with a brush impregnated with silicon carbide powder (Brushine), using constant pressure without paste.
	Intra Oral Diashine^®^ polishing compound	VH Technology (Seattle, WA, USA)	
	SHP Soft Bristle Brush	VH Technology (Seattle, WA, USA)	
	Brushine	Coltène-Whaleden, (Altstätten, Switzerland)	
Polishing protocol No. 4 (P4) (control)	TDF series 014—extra-fine diamond bur Batch: 97529	NTI-Kahla GmbH, (Kahla, Germany)	Surface finishing with extra-fine diamond bur—TDF

* According to the SDS, the Intra Oral Diashine paste is composed of diamond powder in oxyalkylene polymer.

**Table 3:** Mean (M) and standard deviation (SD), expressed as M (±SD), and 95% confidence interval for mean (95% CI) of surface roughness (Ra, nm) for the different resin composites as a function of the polishing protocol (P1-P4) (*n* = 5, for each group).

	Filtek™ Bulk Fill	Fill-Up™	Filtek™ Z250
Polishing Protocol	M (±SD) [95% CI]	M (±SD) [95% CI]	M (±SD) [95% CI]
**P1**	142.4 (±28.9) ^aA^ [106.5-178.3]	304.5 (±31.0) ^aB^ [266.1-343.0]	111.2 (±18.1) ^aC^ [88.7-133.6]
**P2**	135.2 (±33.8) ^aA^ [93.1-177.2]	213.0 (±29.5) ^bB^ [176.3-249.7]	147.8 (±32.2) ^aA^ [107.9-187.7]
**P3**	40.8 (±18.7) ^bA^ [17.6-64.0]	218.8 (±16.0) ^bB^ [199.0-238.7]	68.1 (±15.2) ^bA^ [49.2-86.9]
**P4 (control)**	223.8 (±19.1) ^cA^ [200.1-247.5]	328.6 (±27.5) ^aB^ [294.4-362.7]	207.2 (±7.8) ^cA^ [197.5-216.9]

Different lowercase letters indicate significant differences between means in the same column and different uppercase letters indicate significant differences between means in the same row (Tukey HSD post-hoc test, *p* < 0.05).

**Table 4:** Mean (M) and standard deviation (SD), expressed as M (±SD), and 95% confidence interval for mean (95% CI) of color change (ΔE, %) for the different resin composites as a function of the polishing protocol (P1-P4) (*n* = 5, for each group).

Polishing Protocol	Filtek™ Bulk Fill M (±SD) [95% CI]	Fill-Up™ M (±SD) [95% CI]	Filtek™ Z250 M (±SD) [95% CI]
**P1**	10.9 (±0.5) ^aA^ [10.3–11.6]	11.2 (±0.8) ^aA^ [10.1–12.3]	10.4 (±1.2) ^aA^ [8.8–11.9]
**P2**	9.2 (±0.8) ^bA^ [8.3–10.2]	11.0 (±0.5) ^aB^ [10.3–11.6]	8.2 (±0.2) ^bC^ [7.9–8.4]
**P3**	8.5 (±0.8) ^cA^ [7.5–9.5]	10.7 (±0.6) ^aB^ [9.9–11.5]	7.2 (±0.4) ^bA^ [6.6–7.7]
**P4** **(control)**	13.2 (±0.6) ^dAB^ [12.4–14.0]	14.6 (±0.4) ^bA^ [10.0–15.1]	12.5 (±0.7) ^cB^ [11.7–13.4]

Different lowercase letters indicate significant differences between means in the same column and different uppercase letters indicate significant differences between means in the same row (Tukey HSD post-hoc test, *p* < 0.05).

**Table 5:** Two-way MANOVA results for surface roughness (Ra) and color change (ΔE), considering the factors resin composite type and polishing procedure.

Source	Type III Sum of Squares	d*f*	Mean Square	*F*	*p*	Partial Eta Squared
Model (corrected) (Ra)	419,501.979	11	38,136.544	63.652	<0.001	0.936
Model (corrected) (ΔE)	254.725	11	23.157	47.867	<0.001	0.916
Resin composite type (Ra)	231,238.345	2	115,619.172	192.975	<0.001	0.889
Polishing procedure (Ra)	159,052.904	3	53,017.635	88.489	<0.001	0.847
Resin composite type × polishing procedure (Ra)	29,210.730	6	4868.455	8.126	<0.001	0.504
Resin composite type (ΔE)	54.000	2	27.000	55.811	<0.001	0.699
Polishing procedure (ΔE)	190.330	3	63.443	131.144	<0.001	0.891
Resin composite type × polishing procedure (ΔE)	10.395	6	1.732	3.581	0.005	0.309
Error (Ra)	28,758.802	48	599.142			
Error (ΔE)	23.221	48	0.484			
Total (corrected) (Ra)	448,260.781	59				
Total (corrected) (ΔE)	277.946	59				

**Table 6:** Estimated mean values for surface roughness (Ra) and color change (ΔE), as a function of resin composite type.

	Filtek™ Bulk Fill	Fill-Up™	Filtek™ Z250
**Ra (nm)**	135.5 ^A^	266.2 ^B^	133.6 ^A^
**ΔE (%)**	10.5 ^A^	11.9 ^B^	9.6 ^C^

Different uppercase letters indicate significant differences between means in the same row (Tukey HSD post-hoc test, *p* < 0.05).

**Table 7:** Estimated mean values for surface roughness (Ra) and color change (ΔE), as a function of polishing protocol.

	P1	P2	P3
**Ra (nm)**	186.0 ^A^	165.3 ^A^	109.2 ^B^
**ΔE (%)**	10.8 ^A^	9.5 ^B^	8.8 ^C^

Different uppercase letters indicate significant differences between means in the same row (Tukey HSD post-hoc test, *p* < 0.05).

## Data Availability

The data presented in this study are available on request from the corresponding author.
